# Genetic effect of an InDel in the promoter region of the NUDT15 and its effect on myoblast proliferation in chickens

**DOI:** 10.1186/s12864-022-08362-6

**Published:** 2022-02-16

**Authors:** Chengjie Wei, Yufang Niu, Bingjie Chen, Panpan Qin, Yanxing Wang, Dan Hou, Tong Li, Ruiting Li, Chunxiu Wang, Huadong Yin, Ruili Han, Huifen Xu, Yadong Tian, Xiaojun Liu, Xiangtao Kang, Zhuanjian Li

**Affiliations:** 1 College of Animal Science and Technology, Henan Agricultural University, Zhengzhou, 450046 China; 2 Henan Key laboratory for innovation and utilization of chicken germplasm resources, Zhengzhou, 450046 China; 3 Farm Animal genetic resources exploration and innovation key laboratory of sichuan province, sichuan agricultural university, Chengdu, China

**Keywords:** NUDT15, Indel, Promoter, Muscle, Primary myoblast, Cell proliferation

## Abstract

**Background:**

Molecular breeding accelerates the speed of animal breeding. Screening molecular markers that can affect economic traits through genome-wide association studies (GWAS) can provide a theoretical basis for molecular breeding. At present, a large number of molecular markers have been screened in poultry research, but few reports on how molecular markers affect economic traits exist. It is particularly important to reveal the action mechanisms of molecular markers, which can provide more accurate information for molecular breeding.

**Results:**

The aim of this study was to investigate the relationships between two indels (NUDT15-indel-2777 and NUDT15-indel-1673) in the promoter region of NUDT15 and growth and carcass traits in chickens and to explore the regulatory mechanism of NUDT15. Significant differences were found in genotype and allele frequencies among commercial broilers, commercial laying hens and dual-purpose chickens. The results of association analyses showed that these two indel loci could significantly affect growth traits, such as body weight, and carcass traits. Tissue expression profiling at E12 showed that the expression of NUDT15 was significantly higher in skeletal muscle, and time-expression profiling of leg muscle showed that the expression of NUDT15 in myoblasts was significantly higher in the E10 and E12 proliferation stages than in other stages. Promoter activity analysis showed that pro-1673-I and pro-1673-D significantly inhibited promoter activity, and the promoter activity of pro-1673-D was significantly lower than that of pro-1673-I. In addition, when NUDT15 was overexpressed or underwent interference in chicken primary myoblasts (CPMs), NUDT15 could inhibit the proliferation of CPMs.

**Conclusion:**

The results suggest that the studied indels in the promoter region of NUDT15 may regulate the proliferation of CPMs by affecting NUDT15 expression, ultimately affecting the growth and carcass traits of chickens. These indel polymorphisms may be used together as molecular markers for improving economic traits in chickens.

**Supplementary Information:**

The online version contains supplementary material available at 10.1186/s12864-022-08362-6.

## Background

Studies of the molecular mechanisms of monogenic traits are widely pursued in the molecular breeding of animals and plants due to the unique genotype–phenotype associations of these traits [1, 2]. Many recent studies in chickens have shown that there is a major quantitative trait locus (QTL) at the end of chromosome 1 (Chr1) [3, 4]. This QTL contributes 14.4% of the genetic variation in growth [5], and it has been proven to be significantly linked to growth-related traits such as body and muscle weights [6–9]. Multiple candidate genes exist in this QTL, including MLNR [10], *retinoblastoma 1* (RB1) [11], and *forkhead box O1* (FOXO1) [12]. Chicken nudix hydrolase 15 (NUDT15, 168,554,754**–**168,558,378) is also located in the growth QTL on Chr1. However, the currently reported candidate genes cannot fully explain the genetic variation in growth contributed by this QTL interval.

Multiallelic sites are produced by a high frequency of gene mutations, and gene mutations are a key factor in the formation of genetic diversity [13]. Within species, genetic diversity underlies species diversity [14]. In addition, correctly accounting for multiallelic sites is important both for the understanding of genetic structures in populations and for the more pragmatic purpose of searching for ‘causative’ disease alleles in individuals and cohorts [15]. The study of multiallelic genes is conducive to the maintenance of biodiversity, and identifying dominant multialleles will help accelerate the cultivation of excellent breeds and the determination of therapeutic targets for many diseases.

NUDT15 is a nucleotide triphosphate diphosphatase that consists of 164 amino acids with a nudix hydrolase domain featuring a conserved nudix box that coordinates catalytic Mg^2+^ [16]. NUDT15 is mainly involved in the metabolism of thiopurine, preventing the integration of damaged thiopurine into DNA, repairing DNA mismatches, maintaining steady-state DNA replication, and participating in multiple cell metabolism pathways [17, 18]. Current research on NUDT15 is mainly focused on the effects of mutations in human NUDT15 on the metabolism of purine drugs, which are widely used in the treatment of leukemia and autoimmune diseases [19–21]. NUDT15 (also known as MYH2) can regulate the replication of DNA by affecting the metabolism of thiopurine, and a decrease in its expression can inhibit the proliferation of NB4 and HL-60 cells [22]. The development of a chemical probe against NUDT15 facilitated the discovery that it can promote cell proliferation by forming a complex with proliferating cell nuclear antigen (PCNA) and inhibiting the degradation of PCNA [23]. Proliferating cell nuclear antigen is protected from degradation by forming a complex with MutT homolog2. However, there has been little research and reporting on NUDT15 in chickens.

Promoters are DNA sequences that regulate gene expression by recognizing and binding to regulatory elements such as transcription factors and enhancers [24, 25]. In recent years, gene transcriptional regulation by promoters in livestock and poultry has been widely studied in the context of disease treatment, species evolution and developmental regulation [26, 27]. Many studies have shown that mutations in the promoter region can play a key role in regulating growth and carcass traits [28–30] and hence can have important impacts on the economic traits of livestock and poultry. Screening for mutations related to development, especially causal mutations, will help accelerate livestock and poultry breeding processes.

This study aimed to explore the effects of indels in the promoter region of NUDT15 on the growth and carcass traits of the F_2_ resource group and on the proliferation of chicken primary myoblasts (CPMs). We found that NUDT15 is located within a QTL on Chr1 that is significantly related to chicken growth and development. We experimentally determined the differences in F_2_ resource group growth and carcass traits between chickens with different genotypes of the NUDT15 promoter-region, NUDT15-indel-2777 and NUDT15-indel-1673, and performed gene overexpression and interference experiments to explore the function of NUDT15 in CPMs. We found that the NUDT15-indel-2777 and NUDT15-indel-1673 genotypes were significantly related to multiple growth and carcass traits. In particular, NUDT15-indel-2777 can regulate the transcriptional activity and gene expression of NUDT15 and thus regulate the proliferation of CPMs. This study aimed to explore the role of NUDT15 in chicken growth and development and to provide useful information for chicken molecular breeding.

## Results

### Genotyping and sequencing confirmation

**Fig. 1:**
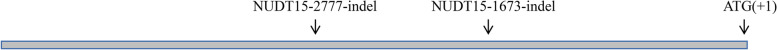
The relative position of two mutation sites. The two indel mutations of 23 bp in the promoter region of the NUDT15 gene, which were located 2777 bp and 1673 bp from the ATG and thus named NUDT15-indel-2777 and NUDT15-indel-1673

**Fig. 2:**
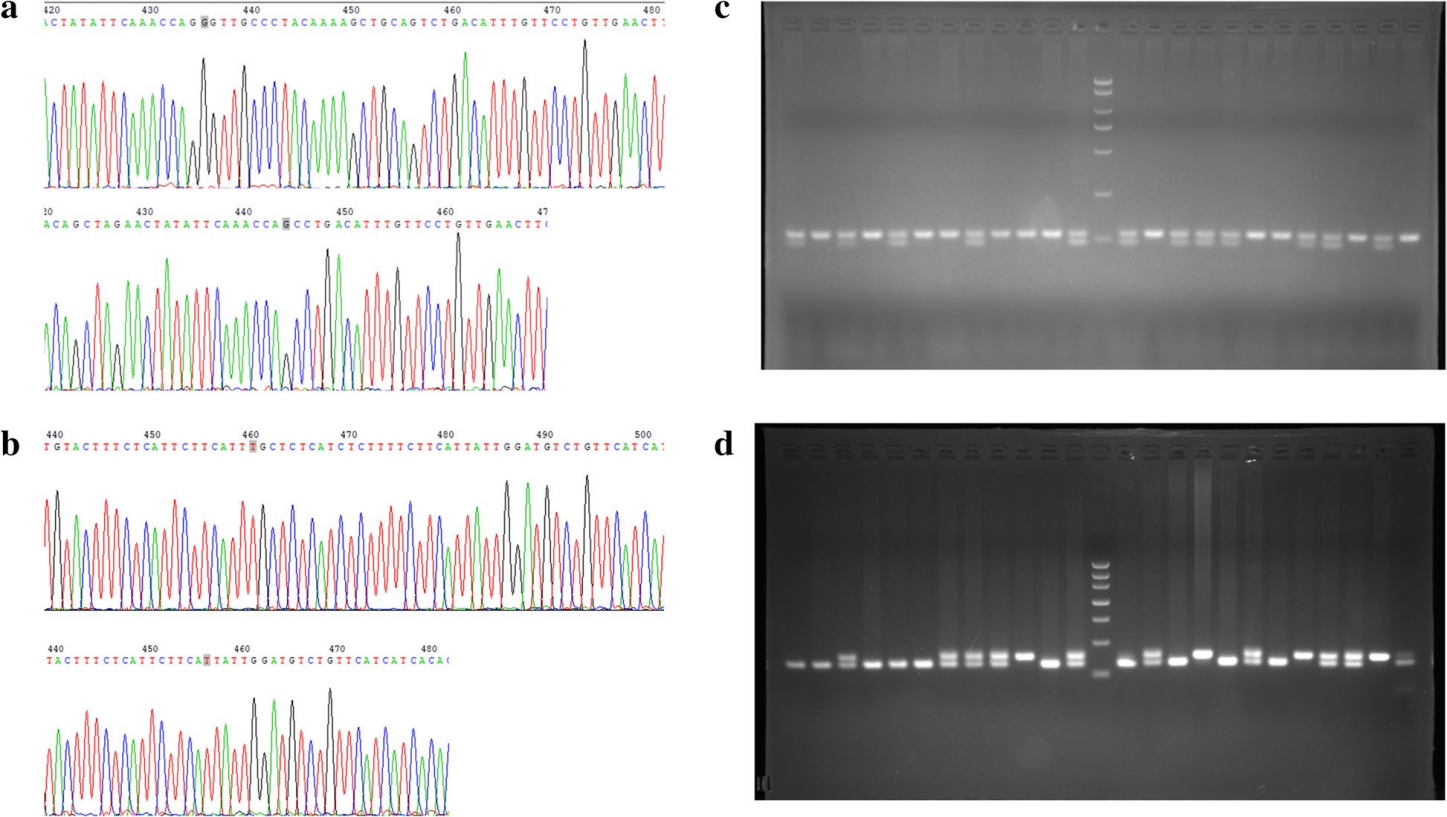
Sequencing comparison of two novel 23-bp indel mutations polymorphism of promoter region within the chicken NUDT15 gene and electrophoresis pattern. **a** NUDT15–2777-indel sequencing comparison. **b** NUDT15–1673-indel sequencing comparison. **c** Electrophoresis pattern of the NUDT15–2777-indel. **d** Electrophoresis pattern of the NUDT15–1673-indel

In this study, the insertion region was sequenced to determine the nature of insertions/deletions. According to the sequencing results, we identified two 23-bp indel mutations in the promoter region of NUDT15, which were located 2777 bp and 1673 bp from the ATG and thus named NUDT15-indel-2777 and NUDT15-indel-1673 (Fig. 1). According to the electrophoresis results, there were two genotypes of NUDT15-indel-2777 (Fig. 2a, b), which were named N^1^ (113 bp) and N^2^ (113 bp/90 bp), and three genotypes of NUDT15-indel-1673 (Fig. 2c, d; the full-length gels are included in S1 and S2), which were named N^3^ (150 bp), N^4^ (127 bp) and N^5^ (150 bp/127 bp).

### Relative expression of NUDT15 in different genotypes and spatiotemporal expression of NUDT15

**Fig. 3:**
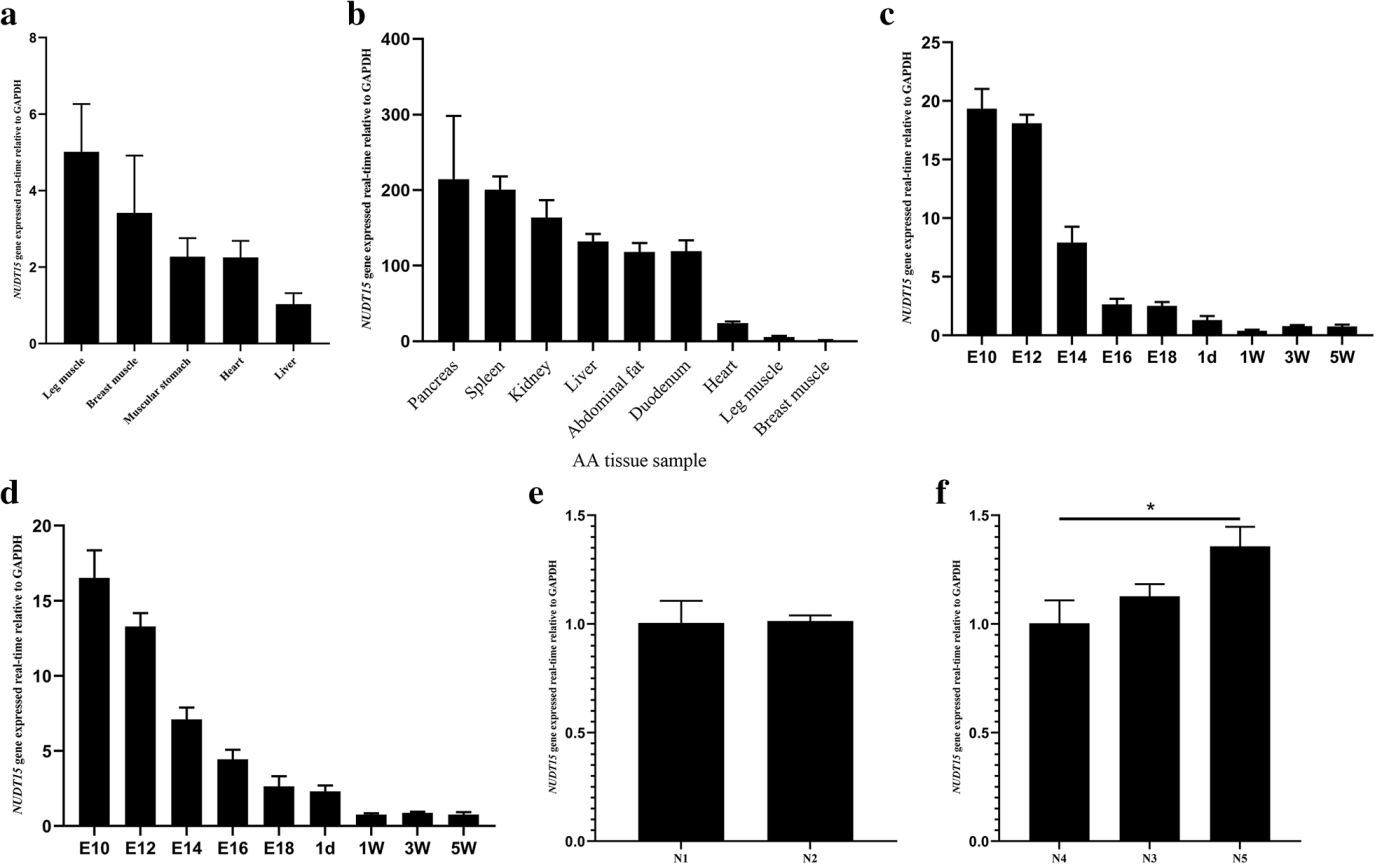
Expression analysis of the NUDT15 gene. **a** Tissue expression profiles of the NUDT15 gene in E10-old AA chickens. **b** Tissue expression profiles of the NUDT15 gene in 2-week-old AA chickens. **c** and **d** Spatiotemporal expression of the NUDT15 gene in leg muscle tissue of AA broiler and Lushi chickens. **e** and **f** Expression pattern of chicken NUDT15 mRNA for various genotypes of NUDT15–2777-indel and NUDT15–1673-indel (*n* = 6). Results are expressed as the mean ± SEM. Statistical significance of differences between means was assessed using independent sample t test. ^∗^*p* < 0.05, ^∗∗^*p* < 0.01

The expression of chicken NUDT15 was detected by quantitative polymerase chain reaction (qPCR) in different tissues and at different developmental stages in AA broilers and LS chickens. The expression of NUDT15 in the leg and breast muscles was significantly higher than that in other tissues in E12-stage AA broilers (Fig. 3a), but the expression in skeletal muscle was significantly lower than that in the pancreas and liver in chicks at 2 w (Fig. 3b). The NUDT15 expression levels in the chest and leg muscles of AA and LS chickens were significantly higher in E10 and E12 than in other periods (Fig. 3c, d). Relative expression analysis in leg muscle isolated from animals with different genotypes showed that NUDT15 expression was not significantly different between the N^1^ and N^2^ genotypes, whereas NUDT15 was expressed at lower levels in N^4^ than in N^3^ and N^5^ (Fig. 3e, f) (*P* < 0.05).

### Genotypic and allelic frequencies of NUDT15-indel-2777 and NUDT15-indel-1673 among the F_2_ resource population and different breeds

**Fig. 4:**
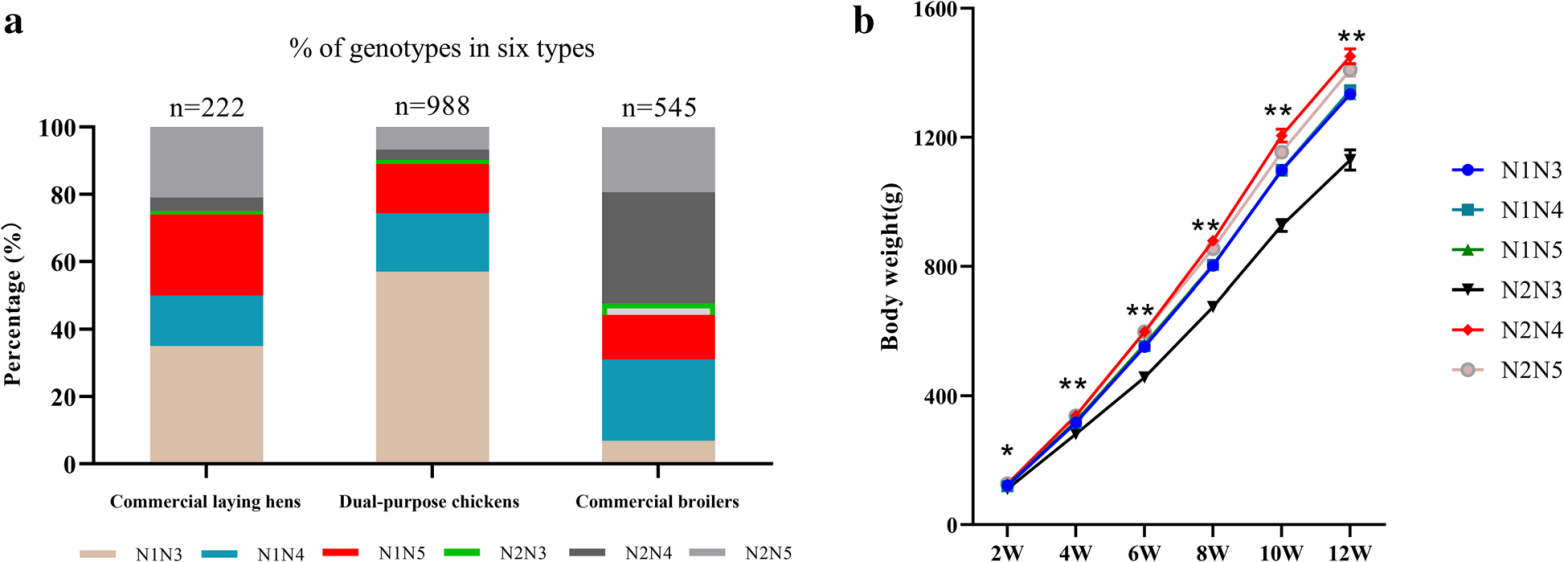
Genotype percentage statistics and growth curves. **a** Percentage of different genotypes in different populations. Commercial laying hens (Hy-Line), dual-purpose type (Xichuan, Guifei, Game fowl, Lushi, Changshun, Gushi),commercial broiler (Hubbard, Arbor Aceres). **b** Developmental changes in body weight of different genotypes of NUDT15 in F_2_ generation at different weeks. Results are expressed as the mean ± SEM. Statistical significance of differences between means was assessed using independent sample t test. ^∗^*p* < 0.05, ^∗∗^*p* < 0.01

We calculated the genotypic and allelic frequencies of NUDT15-indel-2777 and NUDT15-indel-1673 in different breeds (Table 1). In the 10 examined breeds, excluding HBD and AA, the allelic frequencies of 2777-I (F_2_ population: 44%, XC: 50%, GF: 50%, CF: 50%, LS: 45%, CS: 46%, GS: 43%, and HL: 43%) and 1673-I (F_2_ population: 29%, XC: 37%, GF: 27%, CF: 37%, LS: 37%, CS: 37%, GS: 33%, and HL: 29%) were significantly higher than those of 2777-D and 1673-D. The allelic frequencies of 2777-I and 1673-D in commercial broilers (HBD: 36% (2777-I), 38% (1673-D); AA: 37% (2777-I), 36% (1673-D)) were significantly higher than those in dual-purpose chickens and commercial laying hens. In addition, we observed different genotypic frequencies in dual-purpose chickens, commercial laying hens and commercial broilers. N^1^N^3^ was significantly more common than other genotypes in dual-purpose chickens and commercial laying hens, while N^2^N^3^ and N^2^N^4^ were the least frequent genotypes, and only three genotypes (N^1^N^3^, N^1^N^4^ and N^1^N^5^) were observed in XC, GF, and CF. Among commercial broilers, N^2^N^4^ was the predominant genotype. In addition, the frequencies of the N^2^N^4^ and N^2^N^5^ genotypes were significantly higher in commercial broilers than in dual-purpose chickens and commercial laying hens (Table 1). The distributions of different genotypes in different populations are more intuitively displayed in Fig. 4a.

**Table 1:** Genotypic and allelic frequencies and related genetic parameters for the chicken NUDT15 gene

Breeds/n		Genotypic distribution						Allelic frequencies			
		N^1^N^3^	N^1^N^4^	N^1^N^5^	N^2^N^3^	N^2^N^4^	N^2^N^5^	2777-I	2777-D	1673-I	1673-D
^1^F2	F2/790	0.37	0.17	0.22	0.04	0.08	0.12	0.44	0.06	0.29	0.21
Dual-purpose chickens	XC/308	0.69	0.21	0.10	0.00	0.00	0.00	0.50	0.00	0.37	0.13
	GF/176	0.43	0.36	0.22	0.00	0.00	0.00	0.50	0.00	0.27	0.23
	CF/87	0.60	0.13	0.28	0.00	0.00	0.00	0.50	0.00	0.37	0.13
	LS/182	0.64	0.12	0.05	0.02	0.08	0.09	0.45	0.05	0.37	0.13
	CS/92	0.58	0.09	0.15	0.02	0.04	0.13	0.46	0.04	0.37	0.13
	GS/143	0.48	0.14	0.10	0.03	0.07	0.19	0.43	0.07	0.33	0.17
Commercial laying hens	HL/222	0.35	0.15	0.24	0.01	0.04	0.21	0.43	0.07	0.29	0.21
Commercial broilers	HBD/236	0.05	0.24	0.14	0.03	0.33	0.22	0.36	0.14	0.12	0.38
	AA/309	0.09	0.24	0.13	0.04	0.32	0.17	0.37	0.13	0.14	0.36

Note: *F*_*2*_^1^F_2_ resource population, *XC* Xichuan black-boned chicken, *GF* Guifei chicken, *CF* Cockfighting, *LS* Lushi green-eggshell chicken, *CS* Changshun green-eggshell chicken, *GS* Gushi chicken, *HL* Hy-Line variety brown chicken, *HBD* Hubbard broiler chicken, *AA* Arbor Aceres

### Associations of the multiallelic indel in the NUDT15 gene with carcass traits

NUDT15-indel-2777 and NUDT15-indel-1673 had a significant effect on SEW, EW, EWR, HW, CLW, DWW, PW, BMW, LeW, LMW, BMWR and CW (*P* < 0.01). Among these traits, EWR was extremely positively correlated with the multiallelic indel (*P* < 0.05). In addition, the results showed that the N^2^N^4^ genotype produced the largest carcass traits, followed by N^2^N^5^, N^1^N^3^, N^1^N^4^ and N^1^N^5^, whereas the N^2^N^3^ genotype produced the least desirable carcass traits (Table 2).

**Table 2:** Associations of combination haplotype with growth traits in F_2_ resource population

Growth Traits	Age, week	Multiallelic genotypes (Mean ± SE)						*P*-Value
		N^1^N^3^	N^1^N^4^	N^1^N^5^	N^2^N^3^	N^2^N^4^	N^2^N^5^	
Body weight, g	2	122.022 ± 1.114	119.72 ± 1.684	122.911 ± 1.456	110.818 ± 1.985	126.573 ± 2.388	127.118 ± 1.994	0.037
	4	316.739 ± 2.66	316.133 ± 3.97	321.386 ± 3.382	281.323 ± 3.9	339.06 ± 5.616	337.809 ± 4.844	0.000
	6	551.885 ± 4.962	554.329 ± 7.521	559.657 ± 6.347	457.169 ± 5.907	598.132 ± 10.589	598.615 ± 8.903	0.000
	8	802.291 ± 7.46	804.802 ± 11.14	805.085 ± 9.37	675.268 ± 8.418	879.645 ± 15.991	855.072 ± 13.312	0.000
	10	1098.281 ± 9.142	1099.133 ± 13.513	1096.967 ± 11.8	926.415 ± 18.48	1205.512 ± 19.729	1154.507 ± 16.283	0.000
	12	1330.255 ± 11.035	1345.827 ± 16.141	1334.701 ± 14.032	1129.838 ± 31.326	1451.111 ± 23.608	1409.609 ± 19.692	0.000
shank circumference, cm	4	2.671 ± 0.011	2.673 ± 0.017	2.691 ± 0.014	2.602 ± 0.03	2.777 ± 0.024	2.744 ± 0.02	0.000
	8	3.384 ± 0.013	3.445 ± 0.019	3.412 ± 0.016	3.268 ± 0.049	3.517 ± 0.029	3.447 ± 0.023	0.000
	12	3.798 ± 0.014	3.861 ± 0.021	3.853 ± 0.018	3.612 ± 0.065	3.937 ± 0.03	3.879 ± 0.025	0.000
chest breadth, cm	12	6.305 ± 0.036	6.177 ± 0.053	6.346 ± 0.046	6.594 ± 0.034	6.581 ± 0.078	6.455 ± 0.065	0.000
sternal length, cm	4	6.174 ± 0.03	6.179 ± 0.044	6.236 ± 0.038	6.032 ± 0.044	6.422 ± 0.063	6.267 ± 0.054	0.000
	8	8.879 ± 0.039	8.909 ± 0.057	8.863 ± 0.049	8.652 ± 0.048	9.123 ± 0.083	9.047 ± 0.069	0.000
	12	10.909 ± 0.039	11.056 ± 0.057	10.911 ± 0.05	10.513 ± 0.064	11.321 ± 0.083	11.154 ± 0.07	0.000
Body length, cm	4	11.336 ± 0.045	11.259 ± 0.067	11.426 ± 0.057	10.797 ± 0.024	11.657 ± 0.095	11.551 ± 0.083	0.000
	8	16.194 ± 0.065	16.227 ± 0.096	16.054 ± 0.081	15.029 ± 0.047	16.631 ± 0.138	16.416 ± 0.116	0.000
	12	19.706 ± 0.058	19.857 ± 0.086	19.629 ± 0.075	18.272 ± 0.002	20.204 ± 0.126	19.893 ± 0.105	0.000
Pelvis width, cm	4	5.101 ± 0.025	5.21 ± 0.037	5.114 ± 0.032	5.106 ± 0.091	5.282 ± 0.053	5.201 ± 0.046	0.01
	8	6.847 ± 0.038	6.946 ± 0.056	6.764 ± 0.048	6.785 ± 0.039	7.048 ± 0.081	6.878 ± 0.068	0.037

Note: ^1^ *P*-Value. The *P* < 0.01 show the difference is very significant; The 0.01 < *P* < 0.05 show the difference is significant

### Associations of the multiallelic indel in NUDT15 with growth traits

NUDT15-indel-2777 and NUDT15-indel-1673 had significant effects on body weight (BW) at 2 w, 4 w, 6 w, 8 w, 10 w, and 12 w (Fig. 4b); PW at 4 w and 8 w (*P* < 0.05); shank circumference, sternal length, and body length at 4 w, 8 w, and 12 w; and chest breadth at 12 w (*P* < 0.01). In addition, the results showed that the N^2^N^4^ genotype produced the most desirable growth traits, followed by N^2^N^5^, N^1^N^3^, N^1^N^4^ and N^1^N^5^, and the N^2^N^3^ genotype produced the least desirable growth traits (Table 3).

**Table 3:** Associations of combination haplotype with carcass traits in F_2_ resource population

^1^Carcase Traits	Mean ± SE						^2^*P*-Value
	N^1^N^3^	N^1^N^4^	N^1^N^5^	N^2^N^3^	N^2^N^4^	N^2^N^5^	
SEW	1084.933 ± 9.474	1090.145 ± 13.942	1090.352 ± 12.126	918.999 ± 13.423	1188.336 ± 20.503	1131.238 ± 17.044	0.000
EW	907.493 ± 8.192	909.492 ± 12.017	908.231 ± 10.546	723.094 ± 18.074	997.488 ± 17.63	949.572 ± 14.738	0.000
EWR	68.081 ± 0.117	67.416 ± 0.169	68.041 ± 0.149	64.039 ± 0.379	68.548 ± 0.252	68.237 ± 0.21	0.018
HW	42.804 ± 0.3	42.943 ± 0.443	43.086 ± 0.387	33.435 ± 0.618	45.083 ± 0.65	44.054 ± 0.54	0.004
CLW	57.085 ± 0.579	58.911 ± 0.857	58.353 ± 0.743	47.902 ± 0.974	63.52 ± 1.254	60.246 ± 1.034	0.000
DWW	121.293 ± 1.102	120.718 ± 1.626	121.298 ± 1.414	107.199 ± 1.272	131.228 ± 2.386	126.806 ± 1.968	0.000
PW	3.267 ± 0.04	3.402 ± 0.058	3.365 ± 0.051	2.279 ± 0.475	3.55 ± 0.086	3.495 ± 0.07	0.002
BMW	68.173 ± 0.857	68.085 ± 1.27	70.335 ± 1.1	63.975 ± 1.285	80.753 ± 1.871	74.627 ± 1.531	0.000
LeW	147.192 ± 1.379	148.177 ± 2.04	147.622 ± 1.776	118.674 ± 2.426	161.028 ± 2.98	155.569 ± 2.458	0.000
LMW	98.157 ± 1.033	96.924 ± 1.532	98.263 ± 1.325	80.475 ± 1.367	107.902 ± 2.209	104.103 ± 1.822	0.000
BMWR	14.955 ± 0.103	14.892 ± 0.152	15.409 ± 0.133	17.748 ± 0.23	16.168 ± 0.224	15.428 ± 0.186	0.000
CW	1170.048 ± 9.697	1175.501 ± 14.284	1176.849 ± 12.424	998.111 ± 16.575	1270.625 ± 20.956	1235.373 ± 17.398	0.000

Note: *SEW* ^1^semi-evisceration weight, *EW* evisceration weight, *EWR* evisceration weight ratio, *HW* head weight, *CLW* claw weight, *DWW* double wings weight, *PW* pancreas weight, *BMW* breast muscle weight, *LeW* leg weight, *LMW* leg muscle weight, *BMWR* breast muscle weight ratio, *CW* carcass weight

^2^ *P*-Value. The *P* < 0.01 show the difference is very significant; The 0.01 < *P* < 0.05 show the difference is significant

### Promoter activity of NUDT15

**Fig. 5:**
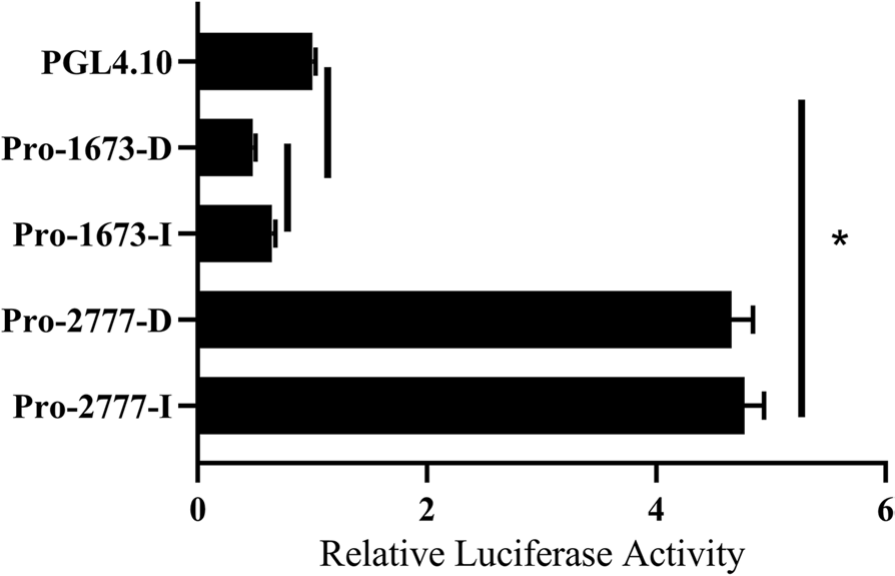
The Promoter Activities of different genotypes of NUDT15(*n* = 3). Results are expressed as the mean ± SEM. Statistical significance of differences between means was assessed using independent sample t test. ^∗^*p* < 0.05

For NUDT15-indel-2777 and NUDT15-indel-1673, mutant-type (I) and wild-type (D) PGL4.10 vectors were constructed and named Pro-2777-I, Pro-2777-D, Pro-1673-I, and Pro-1673-D. Fluorescence activity measurements of Pro-2777-I and Pro-2777-D showed that the activities of Pro-2777-I and Pro-2777-D were significantly higher than that of pGL4.10. Moreover, the promoter activity of Pro-2777-I was higher than that of Pro-2777-D, but the difference was not significant. Fluorescence activity measurements of Pro-1673-I and Pro-1673-D showed that the activities of Pro-1673-I and Pro-1673-D were significantly lower than that of pGL4.10. Additionally, the promoter activity of Pro-1673-I was significantly higher than that of Pro-1673-D (Fig. 5).

### NUDT15 inhibited CPM proliferation

**Fig. 6:**
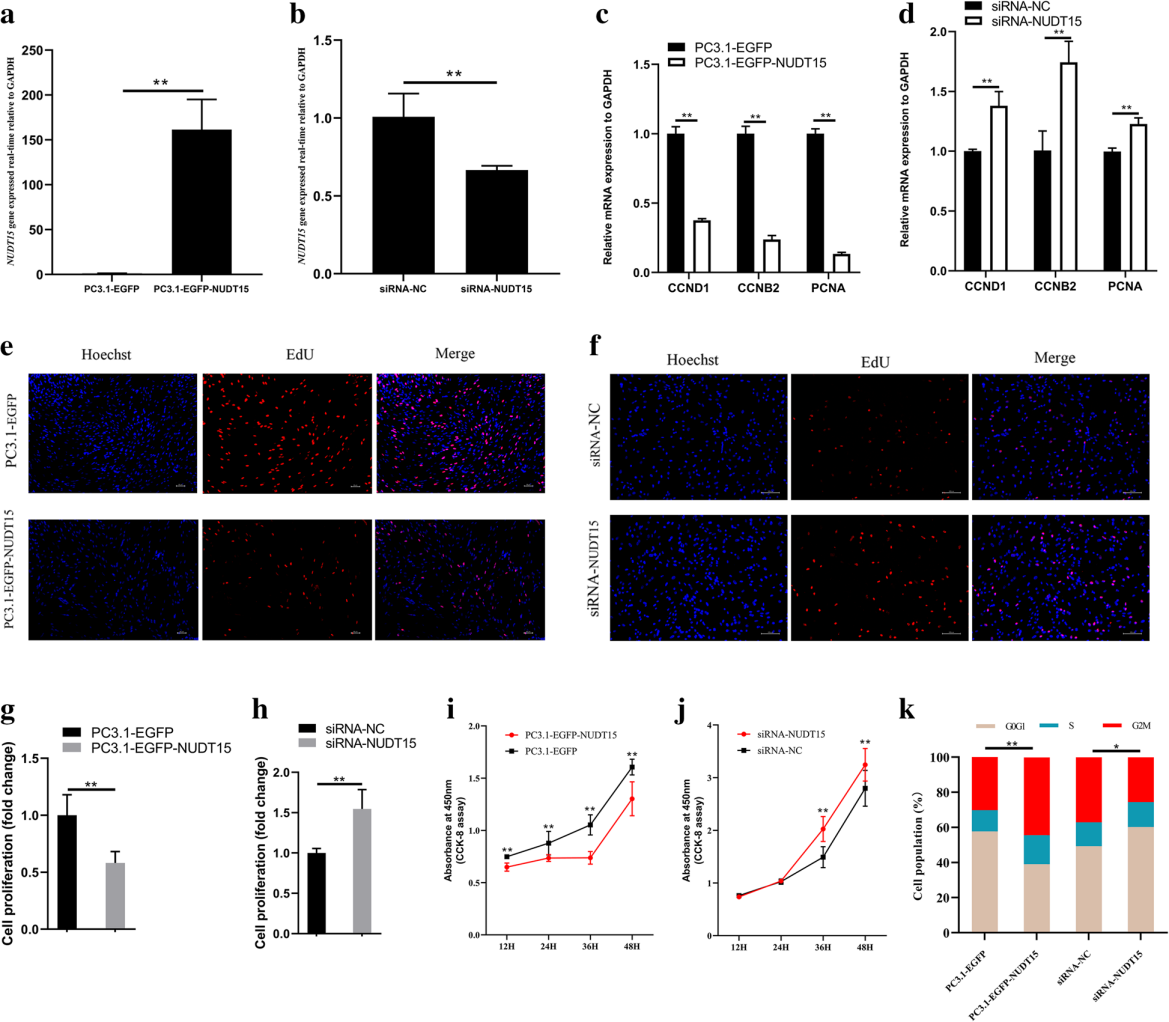
The Effect of lnc9141 on Myoblast Proliferation. **a**-**k** When overexpressing or inferring with NUDT15, respectively, (**a** and **b**) mRNA expression of NUDT15 were detected (*n* = 6); (**c** and **d**) mRNA expression of CCND1, CCNB2, and PCNA were detected (*n* = 6); (**e**-**h**) the number of proliferation cells was counted (*n* = 3); (**i** and **j**) Cell growth was measured after NUDT15 overexpression or inferring (*n* = 8); (**k**) Cell cycle analysis of CPMs with NUDT15 overexpression or inferring (*n* = 3)

To explore the effect of NUDT15 on CPMs, we transfected these cells with a NUDT15 overexpression vector or small interfering RNA (siRNA). The transfection efficiency was analyzed by qPCR, which showed that both the overexpression vector and siRNA significantly changed the expression of NUDT15 (Fig. 6a, b). The expression of cell cycle-promoting genes, including cyclin D1 (CCND1), cyclin B2 (CCNB2), and PCNA, was significantly decreased upon overexpression of NUDT15 (Fig. 6c). After NUDT15 siRNA interference, the expression of these genes increased significantly (Fig. 6d). Using 5-ethynyl-2′-deoxyuridine (EdU) and Cell Counting Kit-8 (CCK-8) assays (Fig. 6e, f, g, h, i, j), we found that NUDT15 overexpression inhibited the proliferation of CPMs. In addition, the overexpression of NUDT15 significantly increased the proportion of cells in G0/G1 phase and decreased the proportions of cells in G2 and S phases. The opposite result was observed after siRNA interference (Fig. 6k). These results suggest that NUDT15 can inhibit the proliferation of CPMs.

## Discussion

Many studies have shown that gene polymorphisms are associated with many important economic traits in chickens. Screening for molecular markers of increased BW and muscle weight and other important economic traits is an important direction in poultry molecular breeding [29–31]. The aim of this study was to determine the effects of indels in the promoter region on growth and carcass traits and on the transcriptional activity of NUDT15 and to determine the mechanisms of the identified indel effects on growth and carcass traits in chickens.

Nudix hydrolase (NUDT) genes, which together comprise the NUDIX gene family, belong to a superfamily of enzymes that are conserved across all species [32, 33]. At present, 18 types of NUDIX proteins have been identified, and all family members except NUDT22 have NUDIX domains. Members of the NUDIX gene family are involved in many biological processes. One biological function in which NUDIX enzymes seem to play a role is cell cycle regulation [34, 35]. NUDT1, NUDT5, and NUDT14 are highly expressed in cancers, indicating a potential role of these NUDIX enzymes in cancer [36]. In addition, most NUDIX proteins act on one to many substrates, which is essential for hormone-induced chromatin remodeling, transcriptional regulation, and cell proliferation [37, 38]. However, previous studies have shown that NUDT15 promotes cell proliferation by binding to PCNA and regulating DNA replication in mammalian studies [23], which is contrary to our determined role in CPMs. In comparing the amino acid series of mammals and poultry, we found great differences, and we speculate that these large differences in amino acids lead to different enzyme functions.

Allelic frequency is used to show the diversity of genes in a population arising through potential genetic drift and the introduction of novel mutations [39]. Studying the genotype distribution and allelic frequencies of gene mutations in different breeds is helpful for understanding their genetic diversity. In this study, two indels in the promoter region of NUDT15 combined to form six genotypes. N^1^N^4^ was the main genotype in meat and egg hens and commercial laying hens, and the N^1^ allele was significantly more common than the other alleles. The proportion of commercial broilers and commercial laying hens was the highest in the N^2^N^5^ genotype, and the proportions of 2777-I and 1673-D in these chickens were significantly higher than those of 2777-D and 1673-I. This may be due to the high degree of manual selection of this breed. Genotypic and allelic frequencies highly differ in different breeds, indicating that NUDT15 is highly polymorphic among different breeds. Further association analysis will be helpful for screening the dominant genotypes of NUDT15.

The results of association analysis showed that indels in the promoter region of NUDT15 could significantly affect the BW of chickens at 2 w, 4 w, 8 w, 10 w, 12 w, as well as the SC, CB, SL, and BL. The phenotypic value of the N^2^N^4^ genotype was significantly higher than those of other genotypes. Previous studies have shown that chicken body shape is related to bone and muscle development. SL can reflect chicken bone and muscle development. A higher SL indicates better bone and muscle development. The growth rate of chickens is highly correlated with carcass traits, and carcass traits can relatively directly reflect the economic value of chickens. The results of the correlation analysis of carcass traits showed that SEW, EW, BMW, LMW and other key economic indicators had higher values in chickens with the N^2^N^4^ genotype. In addition, the expression of NUDT15 in the leg muscle of AA broilers and LS chickens decreased gradually over time, indicating that NUDT15 may be involved in leg muscle development. This observation lays a foundation for further studies of the regulatory mechanisms of NUDT15 affecting skeletal muscle development.

Pro-1673 inhibited the expression of the NUDT15 gene, and the promoter activities of different genotypes were significantly different. The results showed that NUDT15-indel-1673 inhibited gene expression by inhibiting the activity of the NUDT15 gene promoter to different degrees. We speculate that NUDT15-indel-1673 may have significant effects on the carcass and growth traits of the F_2_ resource population through this mechanism. Pro-2777 promoted the expression of the NUDT15 gene, but there were no significant differences in promoter activity among different NUDT15-indel-2777 genotypes. NUDT15-indel-2777 may regulate differences in growth and carcass traits among different genotypes in other ways. Reducing the expression of the NUDT15 gene could promote the proliferation of CPMs, but the molecular mechanism of its inhibition is not clear. NUDT15 is highly expressed in the embryonic stage, and further analysis of the causes of the observed phenomena, the mechanisms by which NUDT15 plays different roles in mammals and poultry need to be further studied.

## Conclusion

In summary, we found two 23-bp indels in the promoter region of NUDT15. Association analysis showed that these indels could significantly affect chicken growth and carcass traits and that the N^2^N^4^ genotype had the highest phenotypic value among all genotypes. The results of statistical analysis showed that the genotypic frequencies of 2777-I and 1673-D were significantly higher in commercial broilers than in other breeds. Through further study, it was found that NUDT15 could inhibit the proliferation of CPMs. The deletion allele of NUDT15-indel-1673 may inhibit the expression of NUDT15 by reducing promoter activity, thereby promoting the proliferation of CPMs to promote muscle synthesis in chickens and resulting in significantly greater phenotypic value of the N^2^N^4^ genotype. In summary, these results explain the regulatory mechanisms of the effects of NUDT15-indel-1673 on several phenotypes, identify a new molecular marker that may be helpful for poultry molecular breeding, and provide a new reference for the study of molecular markers.

## Materials and methods

### Laboratory animals and data collection

The animal population and experimental data used for association analyses were from the F_2_ resource population constructed by the Poultry Germplasm Resources Innovation Center of Henan Agricultural University [40]. Chickens were euthanized by cervical dislocation. Before euthanized, pentobarbital sodium was injected intravenously at a dose of 30 mg/kg to anesthetize the chickens.

To investigate whether this genotypic variation was present in other breeds of chickens, genomic DNA samples were obtained from 988 healthy individuals belonging to 6 dual-purpose chicken breeds Cockfighting chickens (CF, *n* = 87), Guifei chickens (GF, *n* = 176), Xichuan black-bone chickens (XC, *n* = 308), Lushi green-shell layers (LS, *n* = 182), Changshun green-eggshell chicken (CS, *n* = 92), and Gushi chickens (GS, *n* = 143)). In addition, commercial laying hens, namely, Hy-Line (HL, *n* = 222), and commercial broilers, namely, the Hubbard broiler (HBD, *n* = 236) and Arbor Acres (AA, *n* = 309), were also tested to verify the prevalence of these mutations in different breeds.

### Genomic DNA extraction and PCR

A DNA extraction kit was used to extract genomic DNA from blood samples, and the quality of the obtained genomic DNA was determined. The primer pairs used in this study are listed in Table 4. All primers were designed using the online primer design tool Primer-BLAST (http://www.ncbi.nlm.nih.gov/tools/primer-blast/) and synthesized by Sangon Biotech Company (Shanghai, China). The NUDT15 primers were used to identify and genotype the indels in the NUDT15. PCR was performed in 10 μl reactions containing 50 ng of DNA, 0.3 μM of each primer, 5 μl of 2 × Taq Master Mix (Q711, Vazyme Biotech Co., Ltd) and 3 μl of double-distilled water. The cycle parameters were as follows: 95 °C for 5 min; 35 cycles of 95 °C for 10 s, 61 °C for 10 s, and 72 °C for 15 s; and finally an additional 5-min extension at 72 °C. The PCR products were separated by electrophoresis on a 2.5% agarose gel stained with DNAGREEN in 1X TAE buffer. The differences in the frequencies among the populations were analyzed according to the χ^2^ test using SPSS 22.0 (Chicago, IL, USA).

**Table 4:** Details of primer pairs for the chicken NUDT15 gene

Primer set	Primer sequence, 5′-3′	Tm, °C	Product size, bp
*NUDT15*-indel-2777-F	TGTGAGCTACACAGCTAGAACT	61	113
*NUDT15*- indel-2777-R	ATTGGAGGGCTTGGCAATCA		
*NUDT15*- indel-1673-F	ACAGGAGCACATAAATCATAGCCT	62	150
*NUDT15*- indel-1673-R	TGGCCAGTATTTTTAAGTGTGATGA		
qPCR*-NUDT15*-F	ACCTGGAGTTCGGGGAGAG	60	145
qPCR-*NUDT15*-R	ATGAGCACGGTGACGTAGTG		
GAPDH-F	GAACATCATCCCAGCGTCCA	55–65	132
GAPDH-R	CGGCAGGTCAGGTCAACAAC		
CCND1 -F	CAGAAGTGCGAAGAGGAAGT	59	188
CCND1 -R	CTGATGGAGTTGTCGGTGTA		
CCNB2-F	CCTCTTCCACTTCACTTCT	59	195
CCNB2-R	CTTTGTACCCCACTTATCA		
PCNA-F	AGCACCAAATCAGGAAAAG	59	177
PCNA-R	GCACAGGAGATGACAACAG		

### Sample collection, RNA extraction, cDNA synthesis, and qPCR methods

The leg and breast muscle were collected from AA and LS chickens at E10, E12, E14, E16, E18, 1D, 1 W, 3 W, and 5 W. The leg muscle, breast muscle, heart, liver, and muscular stomach of AA at E12 and the leg muscle, breast muscle, heart, liver, pancreas, spleen, kidney, abdominal fat, and duodenum of AA at 2 W were also collected. Total RNA was extracted using RNA isolater Total RNA Extraction Reagent (R401–1, Vazyme Biotech Co., Ltd) as recommended by the supplier. cDNA synthesis for mRNA was carried out using the HiScript III RT SuperMix for qPCR (+gDNA wiper) (R323–01, Vazyme Biotech Co., Ltd). qPCR was performed on an ABI 7500 instrument (Applied Biosystems) using SYBR Green qPCR Mix (Q711, Vazyme Biotech Co., Ltd) according to the manufacturer’s protocol in a two-step method. For each 10 μL reaction, 0.4 μL of 10 μM primer mix and 25 ng of cDNA were added, and 2^−ΔΔCt^ analyses were performed as described previously. Chicken GAPDH was used as an internal control. The primer sequences for all PCRs performed are listed in Table 4.

### Statistical analysis

The growth and carcass traits of the F_2_ population were analyzed by SPSS software (SPSS for Windows, standard version 24.0; SPSS, USA). Two models were used for analysis. Model I was used to evaluate growth traits, and model II considered CW as an adjoint variable. The models were as follows:

- Model I: Y_ijklm_ = μ + G_i_ + S_j_ + H_k_ + f_l_ + e_ijklm_
- Model II: $${\mathrm{Y}}_{\mathrm{i}\mathrm{jklm}}=\upmu +{\mathrm{G}}_{\mathrm{i}}+{\mathrm{S}}_{\mathrm{j}}+{\mathrm{H}}_{\mathrm{k}}+{\mathrm{f}}_{\mathrm{l}}+\mathrm{b}\ \left({\mathrm{W}}_{\mathrm{i}\mathrm{jklm}}\hbox{-} \overset{\hbox{-} }{\mathrm{W}}\right)+{\mathrm{e}}_{\mathrm{i}\mathrm{jklm}}$$

where Y_ijklm_ represents the observed value; μ is the overall population mean; G_i_ represents the fixed effect of the genotype; S_j_ represents the fixed effect of sex; H_k_ represents the fixed effect of the hatch; f_1_ is the fixed effect of the family; e_ijklm_ is the random error; b is the regression coefficient for CW; W_ijklm_ represents the individual slaughter weight; and $\overline{\mathrm{W}}$ is the average slaughter weight. A *p*-value < 0.05 was considered statistically significant, and the influence of the genotypes of the studied polymorphisms on the target traits was investigated by least squares analysis. Bonferroni’s test was used to control for multiple comparisons [41, 42].

### Vector construction

Full-length NUDT15 constructs were cloned into vectors to generate expression plasmids. NUDT15 was cloned into pcDNA3.1 using HindIII/EcoRI. NUDT15 promoter deletion constructs were cloned into pGL4.10 using KpnI/SacI. A ClonExpress II One Step Cloning Kit (C112–02, Vazyme Biotech Co., Ltd) was used to ligate the cloned fragments into each vector.

### Cell culture, transfection, and treatment

DF1 cells were cultured in F12 DMEM (BI) supplemented with 10% FBS (BI) and 1% penicillin/streptomycin (Solarbio).

CPMs were isolated from the leg muscles of embryonic day 11 (E11) individuals as previously described [43]. The obtained cells were cultured in high-glucose DMEM (Biological Industries,Israel) supplemented with 15% FBS (Biological Industries,Israel) and 1% penicillin/streptomycin (Solarbio). For CPM differentiation, CPMs were transferred into differentiation medium supplemented with 2% horse serum when the cells had reached > 90% confluence. Cells were incubated at 37 °C in an atmosphere of 5% CO_2_.

PGL-4.10 and PCDNA3.1-EGFP were transfected using Lipofectamine 3000 (Invitrogen) according to the manufacturer’s protocol into cells at 80% confluence. siRNA-NUDT15 was transfected using Lipofectamine RNAiMAX (Invitrogen) according to the manufacturer’s protocol into cells at 40% confluence.

### Dual fluorescence detection

DF1 cells were cultured in 24-well plates. When the cells reached 80% confluence, they were cotransfected with different recombinant plasmids or the internal reference plasmid (pRL-TK). The total amount of transfected DNA per well was 880 ng, and The transfection ratio of pGL4.10-Pro and the internal reference vector (pRL-TK) was 10:1. Three biological replicates were performed for each treatment. After 36 h, the transfected cells were lysed in 100 μL of 1× passive lysis buffer (PLB) (Promega) at room temperature for 30 min. Dual luciferase activity was measured, and firefly luciferase activity was normalized to Renilla luciferase activity (pRL-TK).

### Flow cytometry, EdU, and CCK-8 assays

For flow cytometry analysis of the cell cycle, myoblasts were seeded in 12-well plates. After a 36-h transfection, cells cultured in growth media were collected and fixed overnight in 75% ethanol at 4 °C. The cells were analyzed with a cell cycle analysis kit (KGA512, KeyGEN BioTECH) on a BD Accuri C6 flow cytometer (BD Biosciences), and the data were processed using FlowJo software (7.6, Tree Star, Ashland, OR, USA).

For the EdU assay, CPMs were seeded in 24-well plates, cultured to 40% density (siRNA) or 70% density (PCDNA3.1-EGFP) and then transfected. Thirty-six hours after transfection, the cells were fixed and stained with an EdU Apollo in vitro imaging kit (C10310, RiboBio) as previously described. A fluorescence microscope (SONY) was used to capture three randomly selected fields, and the number of EdU-stained cells in the fields was determined.

For the CCK-8 assay, CPMs were seeded in a 96-well plate and cultured in growth medium. After transfection, the proliferation of the cell culture at 12, 24, 36, and 48 h was monitored using the CCK8 kit (A311–01, Vazyme Biotech Co., Ltd) according to the manufacturer’s protocol. The absorbance data at 450 nm were read by an iMark microplate absorbance reader (Bio-Rad). All of the data were acquired by averaging the results of six independent replicates.

## Supplementary Information

**Additional file 1.**

## Data Availability

The raw sequence data of NUDT15-indel-2777 and NUDT15-indel-1673 have been deposited at the European Variation Archive (EVA) under Project identified PRJEB47912.
