# Supplementary figures and images for: Genetic effect of an InDel in the promoter region of the NUDT15 and its effect on myoblast proliferation in chickens
Source: BMC Genomics. 2022 Feb 16;23:138. doi: 10.1186/s12864-022-08362-6 (PMC8848950; doi:10.1186/s12864-022-08362-6)

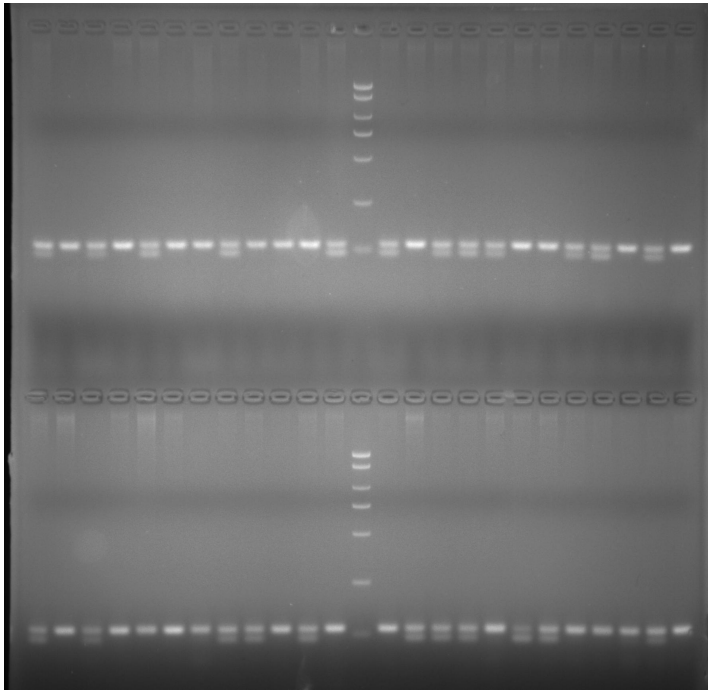

Figure S1. The full-length gels of figure 2c.

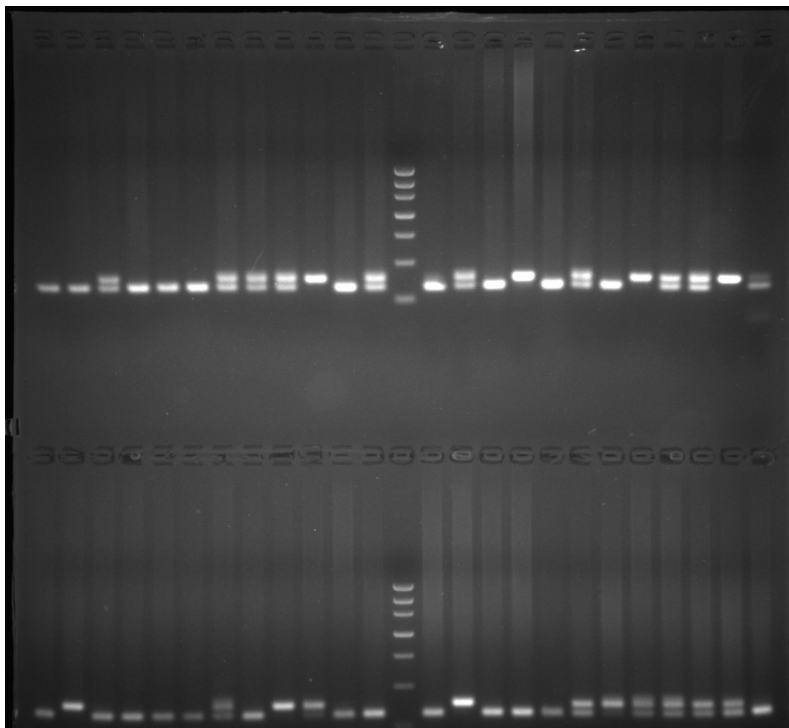

Figure S2. The full-length gels of figure 2d.

Supplement: Supplementary file 1 — Additional file 1. [file 12864_2022_8362_MOESM1_ESM.pdf]
